# Does Hydrate of Chloral Sometimes Act with Unusual Energy When Administered in Conjunction with a Preparation of Opium?

**Published:** 1878-02-20

**Authors:** S. S. Shields

**Affiliations:** Crawfordville, Ga.


### SOUTHERN MEDICAL RECORD.

VOL. VIII. ATLANTA, GA., FEBRUARY 20, 1878. NUMBER 2.

EDITORS:

THOMAS S. POWELL, M. D. W. T. GOLDSMITH, M. D. R. C. WORD, M. D.

*R. C. WORD, M. D., Managing Editor.*

SUBSCRIPTION—$2.00 PER ANNUM, IN ADVANCE.

☞ All Communications and Letters on Business connected with THE RECORD, for 1877 and 1878, must be addressed to the Managing Editor.

### *ORIGINAL AND SELECTED ARTICLES.*

### DOES HYDRATE OF CHLORAL SOMETIMES ACT WITH UNUSUAL ENERGY WHEN ADMINISTERED IN CONJUNCTION WITH A PREPARATION OF OPIUM?

BY S. S. SHIELDS, M.D., Crawfordville, Ga.

Some months since, I was treating a patient for chronic metritis, and during the time, a circumstance occurred which was so unlooked-for and peculi.r that I presume a description of it may not be uninteresting to some of the readers of the RECORD.

I do not propose to give a history of the case, for there is nothing of unusual interest in it. I will state, however, that the patient, previous to my treatment, had begun the use of chloral, and, when she came to me, was taking from 40 to 60 grains daily. This habit I finally succeeded in breaking off, but, in spite of my vigilance, she took to using opium, and was soon under the yoke of that habit. [By way of justice to her, I will say that she suffered greatly all the while, and I gave her some opium during treatment, but did my best to guard against engendering a habit.]

I give the above particulars of her general condition in order that what follows may be better understood.

During the time she was under my care, my patient had several attacks of hysteria, and it was in one of those attacks that what I am about to relate took place.

This attack was an unusually severe one. She had not slept for some fifty or sixty hours. Her appetite was gone, but she would take liquid food mechanically. She began to grow very weak, and active delirium had been developed. I had tried in vain to produce sleep (for that was the main object now); I had gone through the whole list of ordinary remedies: bromide potassium, valerian, ether, assafœtida, cold and hot baths, etc. (I mean head and foot baths.) I had used all means I could think of, both direct and indirect, but they proved of no avail. She gradually grew worse—and right here let me remark how unfortunate it was that she was using opium, for, besides doing no good, it very often does harm in hysteria. It was impossible, however, to take it away from her at this juncture, for, of course, bad results would have surely followed the cutting off of the accustomed stimulant.

One night, about nine o'clock, when I had about got to "my row's end," and she was still delirious, still sleepless, her pulse 120°, respiration 24, yet no fever, skin covered with profuse clammy perspiration, and lips and tongue dry and hot, I concluded to give her a little chloral—though I had almost determined never to give her another dose—not, however, because I had ever seen any *immediate* bad effects from it in her case, but because it had injured her by gradually sapping the strength of her nervous system. I am confident, too, that it had induced organic brain trouble, and that this very trouble was underlying the hysteria at this time.

But to the point: I concluded to give her a little chloral, and accordingly put 10 grains in water and gave her—observe the smallness of the dose. It was my intention to get her gradually under the influence of the remedy, giving her small doses as they were indicated. I proceeded very cautiously, she was so very much prostrated, and it was well that I did, for—mark what followed—I must not forget here to call attention to the fact that she had taken, an hour or two previously, 6 grains of opium (gum). I remained with her, after giving the dose, to watch the effect, and in about fifteen or twenty minutes, after having been awake for fifty or sixty consecutive hours, she fell suddenly into a most profound sleep. The breathing became stertorous, then interrupted and tremulous; her face turned alarmingly pale, and assumed a cadaverous hue, and in less than ten minutes from the time she fell asleep, *respiration ceased;* her muscles became rigid, and the heart beat very rapidly and very feebly. In fact, she exhibited every symptom of chloroform poisoning—that is, the manner in which it poisons in eight cases out of ten—paralysis of the respiratory muscles.

You can imagine my feelings—especially as there was a number of *old women* in the room; but I cannot describe them. But no time was to be lost. I began artificial respiration, slapping with wet towels, etc., and sent immediately for my battery, which, luckily, was not far off, I being in town. It was brought in less than ten minutes, and I began with electricity, placing the positive on the tongue and the negative over the region of the upper portion of the lungs—in fact, as near the pneumo-gastric nerve as I could get it. I then gave her the very strongest (interrupted) current which the instrument was capable of uttering, and had the happiness to see respiration resumed, though, at first, it was only about six to the minute. I kept up the electricity constantly; though it produced most fearful contortions of the muscles, yet it brought the color back to her almost lifeless cheeks, and the respiratory movements gradually increased in rate of speed, until, after five hours of its steady application, she was breathing regularly at seventeen per minute. Her pulse, from beating, scarce perceptibly, at 170° to 180°, came down to its usual rate before giving chloral, namely, 120°.

In the meantime, I had injected, hypodermically, both caffeine and atropia. I gave, during the time, 2 grains of the former (at two doses) and 1-50 grain of the latter. This was done for the reason that I noticed the pin-hole pupil, and, fearful that I should have the accumulated force of the opium to deal with, I began with antidotes for that, for what she had taken during all day (three doses) may have lain unabsorbed in the stomach until the chloral got control of the system, and then the whole amount may have been absorbed and acted upon the nervous centers at once. I say *may have*, because I am not *certain* about opium poisoning. The dose mentioned was not a large one for her. Those who have watched the habits of the opium-eater know how thoroughly used the system becomes to the drug, and with what impunity he can increase his doses. I am certain it was the chloral which first effected her so alarmingly; but why did so small a dose have such an energetic effect? There was no mistake in the weight; she had taken the drug before in four, yes six times the quantity. Then the books say that 47 grains is the lowest poisonous dose yet recorded. As for the sample, I had given some of the same to other patients, and in larger doses. Indeed, I am almost certain that she herself had taken some of it six or eight months before, yet it had been kept well protected.

I have been giving chloral since 1870, and though I never saw such alarming effects from it in any previous case, yet I deem it a very unsafe medicine. It will not act on two persons alike in the same dose, and it will not effect the same person, in the same dose, at different times, alike. There is no certainty in its action. In many instances, it will not produce sleep, unless every other circumstance is favorable.

I see, in the RECORD, a notice of its peculiar effect when given after alcoholic stimulants, or with them. I, too, have noticed a similar effect. It produces intense turgescence of the facial integument, violent throbbing of the heart and arteries, and, in many instances, a maddening urticaria, with singing sound in the ears, etc. I think great danger attends its administration in alcoholismus. Though it may act tolerably, and even very well, in a small number of cases, yet it will deceive you if you do not watch it closely. In fact, it is nót trustworthy, and a remedy that is so potent for evil should never be in the hands of the people at large, as we know this drug is.

Messrs. Editors, if you think the above worthy of a place in your valuable journal, you can put it there. I submit it with the hope that you, or some one oi your correspondents, can advance some argument, *pro.* or *con.*, on the subject. I would like to get the views of some one, more fit than myself, to explain the why and wherefore of the matter.—[We hope that some of our medical brethren will respond.—ED.]

